# The Association between Obstructive Sleep Apnea and Metabolic Markers and Lipid Profiles

**DOI:** 10.1371/journal.pone.0130279

**Published:** 2015-06-26

**Authors:** Wei-Te Wu, Su-Shan Tsai, Tung-Sheng Shih, Ming-Hsiu Lin, Tzu-Chieh Chou, Hua Ting, Trong-Neng Wu, Saou-Hsing Liou

**Affiliations:** 1 National Institute of Environmental Health Sciences, National Health Research Institutes, Miaoli, Taiwan; 2 Graduate Institute of Life Sciences, National Defense Medical Center, Taipei, Taiwan; 3 Institute of Labor, Occupational Safety And Health, Ministry of Labor, Taipei, Taiwan; 4 Department of Public Health, China Medical University, Taichung, Taiwan; 5 Department of Physical Medicine and Rehabilitation, Chung-Shan Medical University, Taichung, Taiwan; 6 Center of Sleep Medicine, Chung-Shan Medical University Hospital, Taichung, Taiwan; 7 Department of Nursing, HungKuang University, Taichung, Taiwan; 8 Department of Public Health, National Defense Medical Center, Taipei, Taiwan; Charité University Medicine Berlin, GERMANY

## Abstract

**Purpose:**

The purpose of this study was to investigate the association between apnea-hypopnea index (AHI) and metabolic markers and whether the elevated risk of Metabolic Syndrome (MetS) is related to Obstructive Sleep Apnea (OSA).

**Methods:**

This cross-sectional study recruited 246 male bus drivers from one transportation company in Taiwan. Each participant was evaluated by a polysomnography (PSG) test and by blood lipids examination. Severity of OSA was categorized according to the apnea-hypopnea index (AHI).

**Results:**

The results showed that a 73.3% prevalence of MetS in OSA (AHI > 15) and a 80.0% prevalence of MetS in severe OSA (AHI > 30) were found. After adjusting for confounding variables, an increased level of Body-Mass Index (BMI) and two non-MetS cardiovascular risk factors, total cholesterol/HDL-C ratio and TG/HDL-C ratio was significantly associated with AHI in subjects with severe OSA. MetS was about three times to be present in subjects with severe OSA, even adjusted for BMI.

**Conclusions:**

The findings showed a high prevalence of MetS in OSA among professional drivers, especially in the severe group category. BMI was the major contributing factor to OSA. However, the present study did not find a sensitive clinical marker of a detrimental metabolic profile in OSA patients.

## Introduction

Obstructive Sleep Apnea (OSA) is a common sleep disorder that is characterized by intermittent, complete, and partial airway collapse, resulting in frequent episodes of apnea and hypopnea [1–3]. The reduction of airflow often leads to acute derangements in gas exchange and recurrent arousals from sleep [3]. Approximately three to seven percent for adult men and two to five percent for adult women in the general population have OSA [4]. OSA is a complex condition and is not limited to a single symptom or feature of the disease [1,2]. Observational and experimental evidence show that OSA contributes to the development of systemic hypertension, cardiovascular disease, and abnormalities in glucose metabolism [1,2,5]. Despite the clinical and scientific advancements in the management of OSA in the last two decades, a great majority (70–80%) of those affected remain undiagnosed [6,7].

The current literature clearly shows that OSA is an emerging risk factor for modulating the cardiometabolic consequences of obesity [2]. In the adult population, the prevalence of OSA is estimated to be as high as 45% in obese subjects [2,8,9]. Metabolic Syndrome (MetS) is a cluster of metabolic abnormalities and is considered as a multi-morbid condition in which the fundamental components are obesity, insulin resistance, hypertension, hypertriglyceridaemia and low high-density lipoprotein cholesterol [10,11]. Moreover, OSA has been associated with heightened metabolic and inflammatory dysregulation, as shown by increases in cytokines [12,13]. The correlates of OSA, including excess body weight and hypertension, overlap with those of diabetes mellitus. OSA was reported to be associated with factors related to MetS [14].

Despite the tight link between MetS and OSA, it is not clear why a significant proportion of OSA patients also have MetS. Moreover, previous studies assess that the overlap between OSA and MetS is simply a result of underlying obesity or unable to adequately assess that OSA represents an additional burden that exacerbates metabolic dysfunction in subjects with MetS. Additionally, patterns of body fat distribution also vary with ethnicity [15,16]. Compared with Caucasians, Asian ethnic groups, e.g., Asian Indians, Chinese, and Japanese have lower values for height, weight,body mass index (BMI), and waist circumference but comparable values for waist–hip ratio and greater body fat [17]. Despite smaller waist circumference in Asian Indians, abdominal fat mass is larger compared with that in Caucasians and African Americans. Moreover, previous studies show that Asian populations are more prone to developing central or abdominal fat, which predisposes them to OSA [1,4]. However, the contribution of OSA to clinical features related to MetS in Asian populations has been seldom investigated.

To assess the contribution of OSA to metabolic markers, the purpose of this study was to investigate (1) the association between apnea-hypopnea index (AHI) and metabolic markers after adjusting for several confounding variables, and (2) whether the elevated risk of cardiovascular diseases including MetS is related to OSA.

## Methods

### Study subjects and data collection

Between May 2007 and July 2008, 1430 professional bus drivers and workers were recruited from one transportation company in Taiwan. The researchers excluded non-professional bus drivers (n = 150) and subjects with no completed the interview questionnaire (n = 295). Among them, 247 subjects agreed to complete a polysomnography (PSG) test at a sleep medicine center. All participants were requested to quit caffeinated diet or beverage after the lunchtime of the experimental day and to arrive at our sleep center at 9:00pm starting with healthy history taking, regular physical examination, Epworth Sleepiness Scale (ESS) and lifestyle questionnaires completing. Based on ESS questionnaire, results showed these participants had symptoms of daytime sleepiness. Sleep was polysomnographically recorded at bedtimes around 10:30 pm-06:00 am. Anthropometric assessments, pre- and post-sleep blood pressures taking and BMI and mean arterial blood pressure calculations were undertaken in our sleep medicine center. Data were not eligible for further processing and analyzing if recorded Total sleep time less than three hours, any measure of anthropometric and testing variables not completed or no written consent forms available.

**Fig 1:**
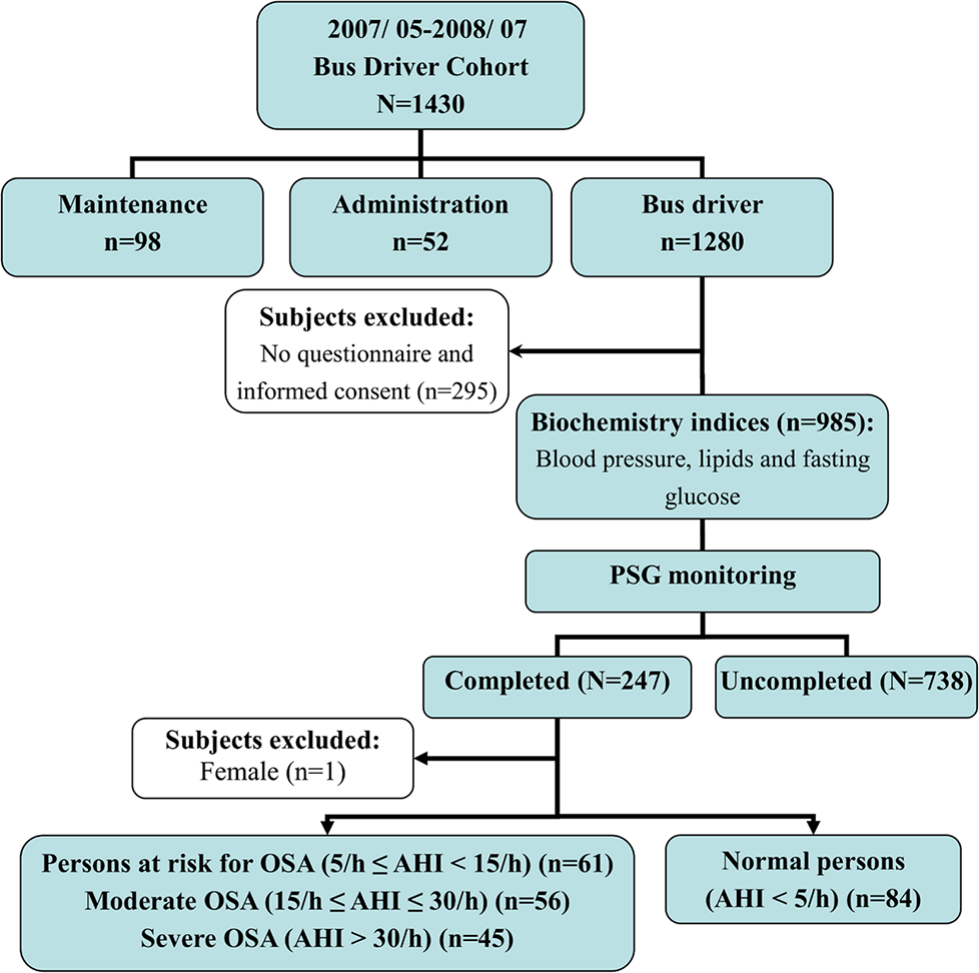
Flow diagram summarized the enrollment of subjects who had undergone polysomnography (PSG).

The researchers excluded 1 female subjects. A total of 246 male bus drivers was included in the subsequent analysis. The study procedures are presented in Fig 1. The comparison with demographic characteristics between completed PSG group (n = 247) and uncompleted PSG group (n = 738) are shown in S1 Table. These two groups are similar in term of gender, marital status, smoking and drinking habits, job types and weekly driving hours. The completed PSG group had older ages, and a higher BMI and education level in comparison with the uncompleted PSG group.

This study was approved by the institutional review board of the National Health Research Institutes, Tri-Service General Hospital, and Chung-Shan Medical University, Taiwan. Informed consent was obtained from each of the subjects after a detailed explanation of the nature and possible consequences of the study were explained by the interviewer on the day of the personal interview. After the written informed consent was obtained from individual participants, the subjects were interviewed in person using a structured questionnaire that included demographics (age, ethnicity, marital and education status), work conditions (work hours and schedule of rotating shifts), lifestyle habits (smoking and drinking), and disease histories. Each participant also underwent a venous blood draw for biochemical screening tests, following a 12-hour overnight fast, during a health examination. The blood samples immediately used for metabolic analysis and enzyme assay.

### PSG and scoring of sleep

A 12-channel polysomnographic recording system (Rembrandt, Medcare, Amsterdam, Netherlands) was used to assess sleep, respiratory and cardiac variables. The recordings of electroencephalography (EEG) (C3/A2, C4/A1), electrooculography, and submental electromyography were used to assess sleep state. These signals were used to determine the sleep stage for each 30 s interval of the polysomnographic record, according to conventional criteria. Oxyhemoglobin saturation (pulse oximetry), nasal airflow, nasal pressure (nasal cannulae and pressure sensor), rib cage and abdominal motion were measured to assess episodes of sleep-disordered breathing. All sleep stages were scored by two well-experienced sleep technologists and further checked by a sleep specialist (Dr. Ting) according to the EEG sleep staging criteria defined by Rechtschaffen and Kales [18].

A drop of airflow by 90% of baseline for at least 10s was defined as an episode of apnea, whereas the oronasal pressure signal excursions drop by 50% of baseline for 10s associated with 3% oxygen desaturation from pre-event baseline or with arousal as hypopnea. Where, AHI was defined as the average number of episodes of apnea and hypopnea per sleep hour. Further, the arousal index (AI) was calculated as the number of EEG arousals per sleep hour. Furthermore, OSA severity is defined as severe OSA (AHI>30 /hr), moderate OSA (AHI: 15–30 /hr), persons at risk for OSA (AHI: 5–14 /hr), and normal persons (AHI<5 /hr) based on AASM consensus report [19].

SaO2 monitoring with a high-resolution pulse oximeter wristwatch (PULSOX-300i, Konica Minolta Sensing, Inc., Osaka, Japan) was performed. Each oxygen probe of the oximeter and PSG were attached to different fingers of the nondominant hand. The sampling frequency of the oximeter PULSOX-300i is 1 Hz on memory interval and an averaging time of 3 seconds. Oxygen Desaturation Index (ODI) is the hourly average number of desaturation episodes, which are defined as at least 4% decrease in saturation from the average saturation in the preceding 120 seconds, and lasting > 10 seconds.

### Blood pressure, lipids and fasting glucose

Two blood pressure recordings (systolic blood pressure and diastolic blood pressure) were obtained from the right arm of subjects in a sitting position after 15 minutes of rest at 5-minute intervals, and their mean value was calculated.

Biochemical analysis of fasting blood glucose (FG) was conducted using Hexokinase method on an AU640 analyzer (Beckman Coulter Ltd., High Wycombe, UK). For the determination of total cholesterol, the assay employed the Cholesterol Oxidase method on an AU640 analyzer (Beckman Coulter Ltd., High Wycombe, UK). Triglycerides (TG) concentration was determined using an enzymatic method on an on an AU640 analyzer (Beckman Coulter Ltd., High Wycombe, UK). High-density lipoprotein cholesterol (HDL-C) level was determined using the immunoinhibition method on an on an AU640 analyzer (Beckman Coulter Ltd., High Wycombe, UK). The ratio of Total cholesterol to HDL-C and TG to HDL-C was calculated.

### Definition of MetS

Participants with MetS were defined by using the criteria proposed by the Taiwan Health Promotion Administration, Ministry of Health and Welfare in 2007 [20]. MetS was defined as the presence of three or more of the following five criteria: (1) waist circumference ≥ 90 cm in men, ≥ 80 cm in women (replaced by BMI >27 Kgs/m^2^); (2) blood pressure ≥130/ 85 mm-Hg or self-reported hypertension; (3) fasting glucose (FG) ≥ 100 mg/dl or self-reported diabetes mellitus; (4) triglyceride (TG) ≥ 150 mg/dl; and (5) high-density lipoprotein cholesterol (HDL-C) < 40 mg/dl in men or < 50 mg/dl in women. Based on the receiver operating characteristic curve analysis in National Nutrition and Health Survey in Taiwan, the BMI of 27 kg/m^2^ can be used in the prediction waist circumference about 90 cm for men and 80 cm for women abdominal visceral obesity [21,22]. We used BMI of 27 kg/m^2^ instead of waist circumference to assess the central obesity in metabolic profile.

### Statistical Analysis

The level of indices exhibited skewed distributions. Therefore, the original data were transformed by using a natural logarithm to approximate a normal distribution. The means and standard deviations were used to describe the distributions of continuous variables. The percentages were used to describe the distributions of categorical variables. Analysis of Variance (ANOVA) was used to test for differences among the four groups, and the test of linearity was used to test for the presence of a linear relationship. Multiple linear regression models were used to test the association between AHI levels and components of MetS after adjusting for age, sex, smoking, drinking, and BMI. Multivariate logistic regression analyses were conducted to evaluate the impact of metabolic markers on OSA. Following those analyses, the hierarchical logistic regression was used to determine the impact of predictors on OSA by using the explained variance (Nagelkerke R Square) from an optimal model. The statistical analyses were performed using SPSS, version 19.0, for Windows. All statistical tests were two-sided with p<0.05 set as the level of statistical significance.

## Results

### Characteristics of Participants

The descriptive statistics of these study participants in terms of demographic characteristics, lifestyle behavior, and job types are presented in Table 1. For the severity of OSA subjects were categorized into four groups: severe OSA group (n = 45; 18.3%), moderate OSA group (n = 56; 22.8%), persons at risk for OSA (n = 61; 24.8%), and normal persons (n = 84; 34.1%). There was no significant difference in the distribution of education status, smoking and drinking habits, and weekly driving hours among the four groups. A trend of an older age and higher BMI was shown to be in agreement with the severity of OSA. Therefore, age and BMI were adjusted in the subsequent analyses.

**Table 1:** Characteristics of study participants assessed by polysomnography.^a^

Variables	Normal persons (n = 84)		Persons at risk for OSA (n = 61)		Moderate OSA (n = 56)		Severe OSA (N = 45)		One-way ANOVA or χ^2^-test	Test of Linearity
	*Mean*	*(SD)*	*Mean*	*(SD)*	*Mean*	*(SD)*	*Mean*	*(SD)*	*p-value*	*p-value*
Age (years)	42.2	(6.7)	43.6	(6.6)	46.8	(7.3)	46.7	(7.8)	<0.001	<0.001
BMI (kg/m^2^)	25.6	(3.0)	27.4	(3.9)	28.9	(4.4)	29.8	(4.1)	<0.001	<0.001
BMI ≥ 30 kg/m^2^; n(%)	6	(7.1)	16	(26.2)	18	(32.1)	16	(35.6)	<0.001	
Neck circumference > 17 inches; n(%)	28	(33.7)	20	(32.8)	28	(50.0)	28	(65.1)	0.002	
	*N*	*(%)*	*N*	*(%)*	*N*	*(%)*	*N*	*(%)*		
Marital status									0.020	
Unmarried	17	(20.2)	6	(9.8)	11	(19.6)	1	(2.2)		
Married	58	(69.0)	52	(85.2)	36	(64.3)	39	(86.7)		
Others	9	(10.7)	3	(4.9)	9	(16.1)	5	(11.1)		
Education									0.239	
≤ Junior high school	20	(23.8)	26	(42.6)	18	(32.1)	17	(37.8)		
Senior high and vocational school	58	(69.0)	30	(49.2)	32	(57.1)	26	(57.8)		
University and College	6	(7.1)	5	(8.2)	6	(10.7)	2	(4.4)		
Cigarette smoking									0.369	
Current smokers	46	(54.8)	33	(54.1)	29	(51.8)	24	(53.3)		
Ex-smokers	5	(6.0)	5	(8.2)	2	(3.6)	7	(15.6)		
Never smokers	33	(39.3)	22	(36.1)	25	(44.6)	14	(31.1)		
Alcohol use									0.769	
Yes	16	(19.0)	9	(14.8)	11	(19.6)	6	(13.3)		
No	67	(79.8)	52	(85.2)	45	(80.4)	38	(84.4)		
Weekly driving hours									0.157	
≥ 90 hours	8	(9.5)	2	(3.3)	1	(1.8)	3	(6.7)		
60–89 hours	54	(64.3)	36	(59.0)	38	(67.9)	22	(48.9)		
≤ 59 hours	22	(26.2)	22	(36.1)	17	(30.4)	20	(44.4)		
Traffic accidents (Year 2006–2009)									0.263	
Yes	10	(11.9)	8	(13.1)	6	(10.7)	1	(2.2)		
No	74	(88.1)	53	(86.9)	50	(89.3)	44	(97.8)		
Heart disease history									0.005	
Yes	4	(4.8)	5	(8.2)	9	(16.1)	11	(24.4)		
No	80	(95.2)	56	(91.8)	47	(83.9)	34	(75.6)		
Hypertension									0.002	
Yes	3	(3.6)	4	(6.6)	8	(14.3)	11	(24.4)		
No	81	(96.4)	57	(93.4)	48	(85.7)	34	(75.6)		
Diabetes									0.174	
Yes	1	(1.2)	2	(3.3)	2	(3.6)	4	(8.9)		
No	83	(98.8)	59	(96.7)	54	(96.4)	41	(91.1)		
Insulin injection									.	
Yes	0	(0.0)	0	(0.0)	0	(0.0)	0	(0.0)		
No	84	(100.0)	61	(100.0)	56	(100.0)	45	(100.0)		
Metabolic Syndrome									<0.001	
Yes	26	(31.0)	32	(52.5)	38	(67.9)	36	(80.0)		
No	58	(69.0)	29	(47.5)	18	(32.1)	9	(20.0)		

^a.^ One-way ANOVA to assess the difference in means and χ2-test to compare frequency distributions

A high percentage of subjects having a history of heart disease (n = 11, 24.4%), hypertension (n = 11, 24.4%) and MetS (n = 36, 80.0%) was found in the severe OSA group in comparison to the others. No significant differences in the distribution of diabetes and hyperlipidemia were found among these four groups.

### Biochemical indices in relation to the severity of OSA

The researchers performed ANOVA to test for differences among the four groups, and the test of linearity to examine for the presence of a linear relationship for blood pressure, blood lipid, PSG, and ODI in relation to the severity of OSA (Table 2). The results showed that the subjects with elevated severity of OSA had a significantly increased trend in metabolic markers including systolic blood pressure, total cholesterol, TG, HDL-C, total cholesterol/HDL-C, and TG/HDL-C and PSG parameters including AI, HI, AHI, and ODI.

**Table 2:** Comparison with blood lipid test, polysomnography, oxygen desaturation index between OSA patients.

Variables	Normal persons (n = 84)		Persons at risk for OSA (n = 61)		Moderate OSA (n = 56)		Severe OSA (N = 45)		One-way ANOVA ^a^ ^.^	Test of Linearity ^a^ ^.^
	*GM*	*(GSD)*	*GM*	*(GSD)*	*GM*	*(GSD)*	*GM*	*(GSD)*	*p-value*	*p-value*
Systolic blood pressure (mmHg)	124.9	(1.10)	126.6	(1.09)	127.3	(1.13)	130.5	(1.11)	0.140	0.024
Diastolic blood pressure (mmHg)	80.2	(1.13)	81.7	(1.13)	81.1	(1.16)	83.7	(1.12)	0.348	0.121
Blood lipid test										
Total cholesterol (mg/dl)	195.9	(1.19)	198.9	(1.17)	202.6	(1.22)	209.3	(1.19)	0.221	0.039
Triglyceride (mg/dl)	162.2	(1.71)	178.6	(1.76)	160.7	(1.72)	210.1	(1.75)	0.051	0.057
HDL cholesterol (mg/dl)	38.5	(1.26)	34.5	(1.23)	35.8	(1.28)	32.0	(1.24)	<0.001	<0.001
Fasting blood glucose (mg/dl)	100.0	(1.44)	101.0	(1.31)	102.2	(1.28)	106.9	(1.46)	0.725	0.289
Total cholesterol/HDL-C ratio	5.09	(1.29)	5.77	(1.30)	5.66	(1.32)	6.54	(1.27)	<0.001	<0.001
Triglyceride/HDL-C ratio	4.21	(1.94)	5.18	(2.01)	4.49	(1.97)	6.56	(1.80)	0.003	0.004
Polysomnography										
Apnea index (AI) (/h)	0.47	(2.31)	2.00	(2.81)	5.74	(3.02)	25.18	(2.31)	<0.001	<0.001
Hypopnea index (HI) (/h)	1.15	(2.30)	5.71	(1.76)	11.15	(1.79)	14.94	(2.33)	<0.001	<0.001
Apnea-hypopnea index (AHI) (/h)	1.59	(2.27)	8.98	(1.36)	21.01	(1.21)	50.33	(1.38)	<0.001	<0.001
Total recording time (TRT) (min)	428.8	(1.08)	430.4	(1.09)	429.1	(1.08)	441.2	(1.10)	0.283	0.137
Total sleep time (TST) (min)	348.5	(1.18)	352.2	(1.16)	351.2	(1.17)	333.9	(1.22)	0.370	0.276
Sleep period time (SPT) (min)	399.2	(1.11)	403.1	(1.11)	404.2	(1.10)	411.8	(1.11)	0.432	0.114
Oxygen desaturation index										
ODI-4% (/h)	2.38	(2.93)	6.46	(2.11)	11.87	(2.34)	17.48	(3.91)	<0.001	<0.001
ODI-3% (/h)	4.05	(2.76)	9.90	(2.09)	16.91	(2.02)	23.79	(2.98)	<0.001	<0.001

^a.^ Assess the difference in mean natural log (Ln)–transformed markers

In comparison with normal persons, severe OSA group was significantly different in TG, HDL-C, total cholesterol/HDL-C, and TG/HDL-C and PSG parameters including AI, HI, AHI, and ODI (post-hoc Scheffe test, P<0.05) (data not shown). Moreover, persons at risk for OSA and moderate OSA group was significantly different in HDL-C, and total cholesterol/HDL-C and PSG parameters including AI, HI, AHI, and ODI when compared to normal persons (post-hoc Scheffe test, P<0.05) (data not shown).

### Associations between AHI and metabolic markers

The multivariate linear regression analyses were conducted to investigate the association between AHI levels and metabolic markers (Table 3). As shown in unadjusted model, BMI, SBP, DBP, HDL-C, total cholesterol/HDL-C, and TG/HDL-C were highly associated with AHI levels. After adjusting for confounders, including age, smoking, and drinking, only BMI significantly associated with AHI levels. Moreover, a significant association between AHI and BMI were noted in BMI < 27 group and BMI ≥ 30 group.

**Table 3:** The association between apnea-hypopnea index (AHI) and each components of metabolic syndrome by BMI.

		Independent variable = LnAHI														
		All subjects (n = 246)						BMI < 27 (n = 128)			27 ≤ BMI < 30 (n = 62)			BMI ≥ 30 (n = 56)		
		Unadjusted			Adjusted			Adjusted			Adjusted			Adjusted		
Model		*β*	*SE*	*p-value*	*β*	*SE*	*p-value*	*β*	*SE*	*p-value*	*β*	*SE*	*p-value*	*β*	*SE*	*p-value*
***Metabolic Syndrome*** ^***a***^ ^.^																
1	BMI	0.044	0.006	<0.001	1.455	0.169	<0.001 ^b^ ^.^	0.335	0.129	0.011 ^b^ ^.^	0.146	0.083	0.084 ^b^ ^.^	1.004	0.402	0.016 ^b^ ^.^
2	Systolic blood pressure	0.01	0.005	0.037	0.001	0.005	0.952^c^ ^.^	0.003	0.008	0.693 ^c^ ^.^	-0.002	0.009	0.862 ^c^ ^.^	0.006	0.015	0.682 ^c^ ^.^
3	Diastolic blood pressure	0.012	0.006	0.049	0.004	0.007	0.556 ^c^ ^.^	-0.001	0.010	0.955 ^c^ ^.^	0.020	0.013	0.120 ^c^ ^.^	-0.014	0.021	0.507 ^c^ ^.^
4	HDL cholesterol	-0.040	0.011	<0.001	-0.009	0.012	0.467 ^c^ ^.^	0.009	0.016	0.581 ^c^ ^.^	-0.026	0.021	0.212 ^c^ ^.^	-0.010	0.033	0.774 ^c^ ^.^
5	Fasting blood glucose	0.004	0.015	0.773	-0.015	0.018	0.404 ^c^ ^.^	-0.020	0.026	0.453 ^c^ ^.^	-0.014	0.031	0.661 ^c^ ^.^	0.004	0.045	0.925 ^c^ ^.^
6	Triglyceride	0.041	0.025	0.104	0.005	0.030	0.862 ^c^ ^.^	-0.037	0.042	0.371 ^c^ ^.^	0.041	0.057	0.468 ^c^ ^.^	-0.024	0.078	0.763 ^c^ ^.^
***Variables not included in MetS criteria*** ^***a***^ ^.^																
7	Total cholesterol	0.012	0.008	0.141	0.010	0.010	0.278 ^c^ ^.^	0.002	0.014	0.895 ^c^ ^.^	0.036	0.017	0.040 ^c^ ^.^	0.006	0.027	0.824 ^c^ ^.^
8	Total cholesterol/HDL-C ratio	0.052	0.012	<0.001	0.019	0.014	0.161 ^c^ ^.^	-0.007	0.018	0.680 ^c^ ^.^	0.062	0.027	0.024 ^c^ ^.^	0.016	0.038	0.681 ^c^ ^.^
9	Triglyceride/HDL-C ratio	0.081	0.031	0.009	0.014	0.350	0.692 ^c^ ^.^	-0.046	0.048	0.337 ^c^ ^.^	0.067	0.065	0.301 ^c^ ^.^	-0.014	0.092	0.878 ^c^ ^.^

^a.^ We used multiple linear regression models to relate natural log (ln)-transformed continuous variables

^b.^ Model adjusted for age, smoking, and drinking.

^c.^ Model adjusted for age, smoking, drinking, and BMI.

After adjusting for such covariables, it was found that ODI-4% and ODI-3% were independently associated with BMI (β = 2.113, p<0.001; β = 2.161, p<0.001). However, ODI (ODI-4% and ODI-3%) and sleep duration (total sleep time (min) and sleep period time (SPT) (min)) had no effect on the other metabolic parameters (data not shown).

### OSA and metabolic markers

The multivariate logistic regression analyses were conducted to investigate the effect of metabolic markers on OSA (Table 4). The presence of severe OSA was a significant factor in an elevated risk of MetS (AOR = 6.904, 95%CI: 2.63–18.14) when compared with the normal persons, after adjusting for age, smoking, drinking, and neck circumference > 17 inches. In Model 2, even additionally adjusted for BMI, the presence of severe OSA also significantly increased the risk of MetS (AOR = 2.920, 95%CI: 1.01–8.43).

**Table 4:** Multiple logistic regression models for odd ratios of components of metabolic syndrome with OSA.^a^

		Persons at risk for OSA vs. Normal persons				Moderate OSA vs. Normal persons				Severe OSA vs. Normal persons			
Model (Dependent variable)		*AOR or β*	*95% CI*		*Nagelkerke R Square (%)*	*AOR or β*	*95% CI*		*Nagelkerke R Square (%)*	*AOR or β*	*95% CI*		*Nagelkerke R Square (%)*
1	Metabolic Syndrome (yes vs. no) ^b^ ^.^	3.677	1.89	7.17		5.568	2.71	11.43		6.904	2.63	18.14	
2	Metabolic Syndrome (yes vs. no) ^c^ ^.^	1.814	0.88	3.74		2.490	1.08	5.73		2.920	1.01	8.43	
***Metabolic Syndrome***													
	BMI ^b^ ^.^												
3	As a continuous	0.100	0.06	0.14		0.146	0.11	0.19		0.162	0.12	0.21	
	As a categorical variable												
4	BMI >27 Kgs/m^2^	3.373	1.79	6.36	6.8 ^d^ ^.^	6.469	3.23	12.94	12.9 ^d^ ^.^	9.350	3.69	23.72	12.2 ^d^ ^.^
5	BMI >30 Kgs/m^2^	7.813	2.93	20.82		9.260	3.43	24.99		9.428	3.08	28.89	
	Systolic blood pressure ^c^ ^.^												
6	As a continuous	-0.003	-0.03	0.03		-0.002	-0.04	0.03		0.160	-0.03	0.06	
7	As a categorical variable												
	SBP ≥ 130 mmHg	0.862	0.45	1.66	0.0 ^d^ ^.^	0.862	0.41	1.81	0.0 ^d^ ^.^	1.386	0.53	3.62	1.1 ^d^ ^.^
	Diastolic blood pressure ^c^ ^.^												
8	As a continuous	-0.006	-0.05	0.03		-0.004	-0.05	0.04		0.023	-0.03	0.08	
9	As a categorical variable												
	DBP ≥ 85 mmHg	1.363	0.70	2.65	0.9 ^d^ ^.^	1.453	0.68	3.09	1.1 ^d^ ^.^	1.529	0.57	4.13	0.4 ^d^ ^.^
	HDL cholesterol ^c^ ^.^												
10	As a continuous	-0.041	-0.11	0.03		-0.028	-0.11	0.05		-0.052	-0.15	0.05	
11	As a categorical variable												
	HDL < 40 mg/dl	1.953	1.01	3.78	3.5 ^d^ ^.^	1.718	0.81	3.66	2.1 ^d^ ^.^	1.888	0.59	6.01	1.7 ^d^ ^.^
	Fasting blood glucose ^c^ ^.^												
12	As a continuous	-0.018	-0.11	0.08		0.016	-0.10	0.14		0.071	-0.10	0.25	
13	As a categorical variable												
	FG ≥ 100 mg/dl	0.948	0.49	1.82	0.0 ^d^ ^.^	1.140	0.55	2.37	0.3 ^d^ ^.^	1.677	0.65	4.32	1.0 ^d^ ^.^
	Triglyceride ^c^ ^.^												
14	As a continuous	-0.054	-0.22	0.11		0.030	-0.16	0.22		0.238	-0.01	0.49	
15	As a categorical variable												
	TG ≥ 150 mg/dl	1.162	0.62	2.18	0.0 ^d^ ^.^	1.507	0.73	3.10	1.0 ^d^ ^.^	2.383	0.89	6.39	3.5 ^d^ ^.^
***Variables not included in MetS criteria***													
	Total cholesterol ^c^ ^.^												
16	As a continuous	0.007	-0.05	0.06		0.048	-0.02	0.11		0.093	0.01	0.17	
	Total cholesterol/HDL ratio ^c^ ^.^												
17	As a continuous	0.048	-0.03	0.13		0.076	-0.01	0.16		0.145	0.04	0.26	
18	As a categorical variable												
	Total cholesterol/HDL ratio ≥ 4.5	1.554	0.73	3.30	1.3 ^e^ ^.^	1.892	0.77	4.62	1.9 ^e^ ^.^	3.094	0.60	16.08	3.3 ^e^ ^.^
	Triglyceride/HDL ratio ^c^ ^.^												
19	As a continuous	-0.013	-0.21	0.18		0.058	-0.16	0.28		0.290	0.00	0.58	
20	As a categorical variable												
	Triglyceride/HDL ratio ≥ 3	1.369	0.67	2.80	0.0 ^f^ ^.^	1.593	0.69	3.70	0.5 ^f^ ^.^	6.322	1.24	32.23	6.3 ^f^ ^.^

^a.^ We used multiple linear regression models to relate natural log (ln)-transformed continuous variables

^b.^ Adjusted for age, smoking, drinking, and neck circumference > 17 inches.

^c.^ Adjusted for age, smoking, drinking, and BMI.

^d.^ The hierarchical regression model was used the predictors including the BMI >27 Kgs/m^2^, SBP ≥ 130 mmHg, DBP ≥ 85 mmHg, HDL < 40 mg/dl, FG ≥ 100 mg/dl, TG ≥ 150 mg/dl, age, smoking, and drinking.

^e.^ The hierarchical regression model was used the predictors including the Total cholesterol/HDL ratio ≥ 4.5, age, smoking, drinking, and BMI.

^f.^ The hierarchical regression model was used the predictors including the Triglyceride/HDL ratio ≥ 3, age, smoking, drinking, and BMI.

No matter what, BMI was evaluated as a continuous or categorical variable. BMI levels significantly associated with OSA in Persons at risk for OSA, Moderate, and Severe categories when compared to normal persons. However, the presence of OSA was not associated with other criteria for MetS after adjustments.

Moreover, there was a strong trend for an independent association between the AHI and total cholesterol/HDL ratio in subjects with severe OSA in comparison with normal persons, even after adjusting for age, smoking, drinking, and BMI. The presence of OSA was also independently associated with the abnormally elevated TG/HDL ratio.

The hierarchical regression model used categorical variables, including the BMI, SBP, DBP, HDL, FG, TG, and cofounder factors including age, smoking, and drinking. The Nagelkerke R^2^ of BMI>27 kg/m^2^ was 6.8% and 12.9% among persons at risk for OSA and moderate OSA subjects when compared with the normal persons. The second contributing factor was HDL-C. Moreover, the Nagelkerke R^2^ of BMI>27 kg/m^2^ was 12.2% among the severe OSA when compared with the normal persons.

## Discussions

This study focused on determining whether OSA had MetS prevalence and whether OSA was independently associated with biomarkers of metabolic dysfunction. The major finding of this study was that there was a 73.3% prevalence of MetS in OSA (AHI > 15) and a 80.0% prevalence of MetS in those with severe OSA (AHI>30). MetS was about three times more likely to be present in subjects with severe OSA, even adjusted for BMI. The elevated BMI and with two non-MetS cardiovascular risk factors, total cholesterol/HDL-C ratio and TG/HDL-C ratio was associated with AHI in subjects with severe OSA in compared with normal persons.

### Prevalence of the MetS

The study design allowed for the researchers to systematically examine the prevalence of unrecognized MetS in subjects with OSA. A 73.3% prevalence of MetS in OSA (AHI > 15) and a 80.0% prevalence of MetS in those with severe OSA (AHI>30) was found. In a Spanish Population study, the prevalence of MetS was higher in OSA patients (43% in the mild-moderate group and 81% in the severe group) than in normal persons (32%) [23]. Ambrosetti et al. studied 89 consecutive OSA patients (AHI ≥ 15) following The US National Cholesterol Education Programme Adult Treatment Panel III (NCEP ATP III) recommendations and found a prevalence of MetS in 53% [24]. In a large series of Mediterranean OSA patients (AHI>5) the prevalence of MetS was between 50.6%-69.8% [25], and in OSA patients (AHI>10) the prevalence of MetS was 51.2% [26]. Moreover, the prevalence rates were reported by clinical studies in Western countries ranging between 30% and 87% [27]. Results showed that the prevalence rate of MetS was higher in severe OSA and mild-moderate OSA subjects than in the above mentioned studies. This is probably due to gender proportions in the study populations or ethnic differences.

### Components of the MetS and OSA

There has been no consistent explanation of the number of components of MetS increases with OSA severity in the previous studies [9,23,26,28]. In this study, researchers found an independent correlation between OSA severity and BMI with two non-MetS cardiovascular risk factors- total cholesterol/HDL-C ratio and TG/HDL-C ratio. In animal models, intermittent hypoxia, a key feature of OSA, is involved in the pathogenesis of an abnormal lipid profile, modifying the expression of lipoprotein lipase. It is key in the HDL-C synthesis and increases the liver content of TG in mice [29,30]. OSA patients have been shown to have increased TG, increased total cholesterol/HDL-C ratio, and lower HDL-C values [31]. They may also have reduced HDL-mediated inhibition of low density lipoprotein oxidation [32]. In many subjects from the Sleep Heart Health study there was a positive association between OSA severity and an increased amount of serum total cholesterol and TG, as well as a decreased amount of serum HDL in subjects under the age of 65 [33]. In hospital populations, severe OSA is associated with low HDL-C levels independently of confusing factors such as obesity [31,34]. Although an independent association between OSA and low levels of HDL-C or total cholesterol/HDL-C ratio seem to be observed in the above mentioned studies and in the present study, the ultimate relationship between OSA and the alterations in lipid metabolism remains to be defined.

### Relationships between MetS, OSA and Obesity

One of the most important associations between MetS and OSA is that obesity is tightly connected. Central, or visceral, obesity is associated with the greatest risk for OSA because fat deposits in the upper airway affect distensibility [35,36]. The increased volume of abdominal fat could predispose one to hypoventilation during sleep and/or reduce the oxygen reserve, leading to oxygen desaturation during sleep [37]. In contrast, nocturnal awakenings and sleep disruption in OSA lead to sleep debt that, in turn, is translated to less activity during the diurnal hours and promotes obesity [38]. The disrupted sleep patterns characteristic of OSA are correlated with metabolic effects and weight gain [38]. These results are consistent with findings from the other studies, in which a correlation between AHI and BMI was found. These also imply that obesity is the major outcome for OSA and leads to MetS.

Moreover, in the present study, MetS was about three times more likely to be present in subjects with severe OSA after adjustment for BMI. Thus, BMI might not fully explain the association between MetS and OSA. It is possible that OSA and MetS share the similar risk factor other than obesity, such as sedentary lifestyle [39]. Further intervention studies will be needed to clarify this by using CPAP or a structured physical activity program.

### Strengths and Limitations

Previous studies seldom address the relationship between MetS and sleep disordered breathing in Asian subjects. An interesting and unexpected observation that has emerged is that the patterns of body fat distribution also vary with ethnicity [15,16]. Although Asians are less obese than Whites, Asians have a greater disease severity than Whites adjusted for age, sex, and BMI [40,41]. Differences in craniofacial features between Asians and Whites have been demonstrated and are considered as etiologic factors for the increased risk and greater severity of OSA in Asians despite lesser degrees of obesity [42].

There were some limitations to this study. First, it is a cross-sectional study. Therefore, results may not be interpreted as having a causal association due to a lack of temporality. Second, the study used BMI of 27 kg/m^2^ instead of waist circumference to assess the central obesity in the metabolic profile. According to Asian-Pacific guidelines for central obesity, optimal waist circumference in Asians is men >90 cm and women >80 cm) [43]. Based on the receiver operating characteristic curve analysis from the National Nutrition and Health Survey in Taiwan, the BMI of 27 kg/m^2^ can be used to predict waist circumference for about 90 cm for men and 80 cm for women abdominal visceral obesity [21,22]. Third, obesity leads to a number of sleep disordered breathing patterns like OSA and obesity hypoventilation syndrome (OHS) at the same time, in particular patients with a BMI over 30 are at risk. This study did not perform arterial blood gas measurement to detect arterial carbon dioxide level. Thus, we did not exclude the possibility that in OHS may be predisposed one to MetS. Fourth, this study did not perform nuclear MRI (NMR) to quantify adipose tissue of the neck regions. Thus, the results do not exclude the possibility that in certain cases increased parapharyngeal fat may be predisposed one to upper airway narrowing and OSA. However, we have adjusted circumference greater than 17 inches to control for alternative explanations. The result still showed that OSA was strongly associated with MetS. Fifth, this study adopted autonomous participation has made it difficult to find a sufficiently large group of bus drivers to category between OSA and MetS. However, the positive association between the AHI and BMI in the BMI category groups was found. In addition, the small numerical, but significant differences of BMI or MetS between those with severity of OSA would be more evident if we had more subjects with OSA. Finally, the inclusion of solely male professional drivers in the study restrict the interpretation of the results in female. Moreover, our outcomes should not be fully extrapolated to general population.

## Conclusion

In summary, the present study showed that OSA was strongly associated with MetS, and the inter-correlation may be involved in the pathogenesis of lipid abnormalities. The study also showed that BMI was the major contributing factor between OSA and MetS. However, the present study did not find a sensitive clinical marker of a detrimental metabolic profile in OSA patients.

## Supporting Information

S1 Table Demographic characteristics of included and excluded subjects. (DOC)
