# Supplementary material for: The Association between Obstructive Sleep Apnea and Metabolic Markers and Lipid Profiles
Source: PLoS One. 2015 Jun 26;10(6):e0130279. doi: 10.1371/journal.pone.0130279 (PMC4483259; doi:10.1371/journal.pone.0130279)
Supplement: S1 Table — (DOC) [file pone.0130279.s001.doc]

| **S1 Table Demographic characteristics of included and excluded subjects** | | | | | | |
| --- | --- | --- | --- | --- | --- | --- |
| **Variables** | **Included (n=247)** | |  | **Excluded (n=738)** | | ***P*-value ^a.^** |
|  | *Mean* | *(SD)* |  | *Mean* | *(SD)* |  |
| Age (years) | 42.4 | (7.2) |  | 40.6 | (7.4) | 0.001 |
| BMI (kg/m^2^) | 27.4 | (4.3) |  | 25.7 | (3.9) | <0.001 |
|  |  |  |  |  |  |  |
|  | *N* | *(%)* |  | *N* | *(%)* |  |
| Gender |  |  |  |  |  | 0.640 |
| Male | 245 | (99.2) |  | 734 | (99.5) |  |
| Female | 2 | (0.8) |  | 4 | (0.5) |  |
| Marital status |  |  |  |  |  | 0.195 |
| Unmarried | 35 | (14.2) |  | 139 | (18.8) |  |
| Married | 185 | (74.9) |  | 511 | (69.2) |  |
| Others | 27 | (10.9) |  | 88 | (11.9) |  |
| Education |  |  |  |  |  | 0.028 |
| ≤Junior high school | 81 | (32.8) |  | 179 | (24.3) |  |
| Senior high and vocational school | 147 | (59.5) |  | 488 | (66.1) |  |
| University and College | 19 | (7.7) |  | 179 | (24.3) |  |
| Cigarette smoking |  |  |  |  |  | 0.680 |
| Current smokers | 132 | (53.4) |  | 415 | (56.2) |  |
| Ex-smokers | 19 | (7.7) |  | 48 | (6.5) |  |
| Never smokers | 95 | (38.5) |  | 272 | (36.9) |  |
| Alcohol use |  |  |  |  |  | 0.846 |
| Yes | 42 | (17.0) |  | 130 | (17.6) |  |
| No | 203 | (82.2) |  | 605 | (82.0) |  |
| Job types |  |  |  |  |  | 0.254 |
| City buses | 22 | (8.9) |  | 93 | (12.6) |  |
| Long-distance Buses (24 hour shift) | 145 | (58.7) |  | 439 | (59.5) |  |
| Long-distance Buses (8 hour shift) | 32 | (13.0) |  | 91 | (12.3) |  |
| Long-distance Buses (others) | 47 | (19.0) |  | 111 | (15.0) |  |
| Weekly driving hours |  |  |  |  |  | 0.305 |
| ≥ 80 hours | 45 | (18.2) |  | 166 | (22.5) |  |
| 60–79 hours | 120 | (48.6) |  | 351 | (47.6) |  |
| ≤ 59 hours | 81 | (32.8) |  | 216 | (29.3) |  |
| ^a^. t-test to assess the difference in means and χ2-test to compare frequency distributions | | | | | | |
